# Modeling Stochastic Variability in the Numbers of Surviving Salmonella enterica, Enterohemorrhagic Escherichia coli, and Listeria monocytogenes Cells at the Single-Cell Level in a Desiccated Environment

**DOI:** 10.1128/AEM.02974-16

**Published:** 2017-02-01

**Authors:** Kento Koyama, Hidekazu Hokunan, Mayumi Hasegawa, Shuso Kawamura, Shigenobu Koseki

**Affiliations:** Graduate School of Agricultural Science, Hokkaido University, Sapporo, Japan; The Pennsylvania State University

**Keywords:** stochastic modeling, single-cell level, survival, variability

## Abstract

Despite effective inactivation procedures, small numbers of bacterial cells may still remain in food samples. The risk that bacteria will survive these procedures has not been estimated precisely because deterministic models cannot be used to describe the uncertain behavior of bacterial populations. We used the Poisson distribution as a representative probability distribution to estimate the variability in bacterial numbers during the inactivation process. Strains of four serotypes of Salmonella enterica, three serotypes of enterohemorrhagic Escherichia coli, and one serotype of Listeria monocytogenes were evaluated for survival. We prepared bacterial cell numbers following a Poisson distribution (indicated by the parameter λ, which was equal to 2) and plated the cells in 96-well microplates, which were stored in a desiccated environment at 10% to 20% relative humidity and at 5, 15, and 25°C. The survival or death of the bacterial cells in each well was confirmed by adding tryptic soy broth as an enrichment culture. Changes in the Poisson distribution parameter during the inactivation process, which represent the variability in the numbers of surviving bacteria, were described by nonlinear regression with an exponential function based on a Weibull distribution. We also examined random changes in the number of surviving bacteria using a random number generator and computer simulations to determine whether the number of surviving bacteria followed a Poisson distribution during the bacterial death process by use of the Poisson process. For small initial cell numbers, more than 80% of the simulated distributions (λ = 2 or 10) followed a Poisson distribution. The results demonstrate that variability in the number of surviving bacteria can be described as a Poisson distribution by use of the model developed by use of the Poisson process.

**IMPORTANCE** We developed a model to enable the quantitative assessment of bacterial survivors of inactivation procedures because the presence of even one bacterium can cause foodborne disease. The results demonstrate that the variability in the numbers of surviving bacteria was described as a Poisson distribution by use of the model developed by use of the Poisson process. Description of the number of surviving bacteria as a probability distribution rather than as the point estimates used in a deterministic approach can provide a more realistic estimation of risk. The probability model should be useful for estimating the quantitative risk of bacterial survival during inactivation.

## INTRODUCTION

There is a risk of infection by foodborne pathogens when certain foods are ingested, even if as few as <10 to 100 Salmonella enterica or enterohemorrhagic Escherichia coli cells are present. Even one of these bacterial cells can cause foodborne disease, although the probability is very low (1). For example, a small number of cells in foods such as chocolate, salami, cheddar cheese, hamburger patties, and raw beef liver have been reported to present a medium risk of causing foodborne illness (2–4). Numerous predictive models based on deterministic approaches focusing on large bacterial populations, for example, more than 10^5^ cells, have been developed to estimate the kinetics of inactivation of pathogenic bacteria (5). However, a deterministic approach results in limited predictions of bacterial behavior when dealing with low numbers of bacteria because this approach does not consider the variability and uncertainty of bacterial behavior (6). In a small population, the effect of the behavior of an individual bacterium becomes relatively large, and individual cell heterogeneity clearly appears when cell numbers are small (5). Because contamination of food with pathogens typically occurs with very low cell numbers, the use of probabilistic approaches that enable a description of the variability of the behavior of single cells is necessary to obtain more realistic estimates of the safety risk (7). Thus, there is a need to develop a predictive model to estimate the behavior of bacteria after the use of inactivation processes at the single-cell level.

In recent years, the need to consider the variability of the various factors that may influence predictive microbiology models has increasingly been recognized and has led to the development of more sophisticated stochastic models (8). Models that predict variability in the behavior of bacteria were developed by incorporating the probability distributions for variability or uncertainty model parameters in a Monte Carlo simulation (5, 6, 9–12). These models incorporated the variability caused by both the microorganism and the environment. Although bacterial behavior appears to fluctuate with low cell numbers, which may represent the natural stochastic variability in bacterial numbers, the randomness of the observed numbers of bacteria has not yet been directly considered or incorporated for evaluating bacterial behavior.

Recently, Koyama et al. (13) developed a sample preparation procedure for the probabilistic evaluation of bacterial behavior by obtaining bacterial numbers following a Poisson distribution (indicated by the parameter λ, which was equal to 2), which represents the variability in the occurrence of a natural event. In their paper, they suggested using the number of bacteria following a Poisson distribution (λ = 2) in a stochastic inactivation approach to investigate the variability in the numbers of surviving bacteria. In a similar approach, the Poisson distribution (λ = 2) was used to investigate the lag phase of single cells (14). To estimate the randomness of the number of bacterial cells that survive a process designed to kill bacteria, which could include heating, desiccation, or acid stress, we considered that the behavior of bacteria after the use of inactivation processes, incorporating the variability in the behavior of individual cells, can be described in a probabilistic model.

Foods with low water activity (*a_w_*; i.e., foods with an *a_w_* of <0.85) do not support the growth of pathogenic bacteria. However, these foods could nevertheless be a vehicle for pathogenic bacteria and cause outbreaks of foodborne illnesses (2, 15, 16). Between 2007 and 2012, there were at least 7,315 cases of bacterial infection and 63 deaths worldwide due to the consumption of contaminated foods with a low *a_w_* (16), and the majority of these outbreaks were associated with S. enterica (2, 15, 17). Infection can result from the consumption of some low-*a_w_* foods, such as chocolate, salami, potato chips, and cheddar cheese, with as few as <10 to 100 S. enterica and enterohemorrhagic E. coli cells (2, 4, 18–20). However, it is still difficult to predict bacterial survival at low levels with current experimental techniques. Estimation of the variability in the number of surviving bacteria will help to provide a more accurate estimate of the risk for contamination of low-*a_w_* foods.

The objective of the present study was to develop a probability model to estimate the variability in the number of surviving bacteria at the single-cell level in an environment with desiccation stress. In particular, we focused on describing the randomness of the small numbers of bacteria that are present because of naturally occurring phenomena and that cannot be eradicated as variability in a Poisson process. We used bacterial cell numbers following a Poisson distribution (λ = 2) to determine the variability in the number of survivors at the single-cell level under desiccation stress at 5, 15, and 25°C. Furthermore, to confirm the validity of the experimentally observed naturally occurring randomness of the bacterial cell numbers in a Poisson process, we examined the bacterial death process using computer simulation. We determined whether a simulated number of survivors followed a Poisson distribution. The probability model developed in this study will enable estimation of the variability in the numbers of surviving bacteria and the probability of survival at the single-cell level.

## RESULTS

### Probability model for estimating the variability in the number of surviving bacteria over time in a desiccated environment.

**FIG 1:**
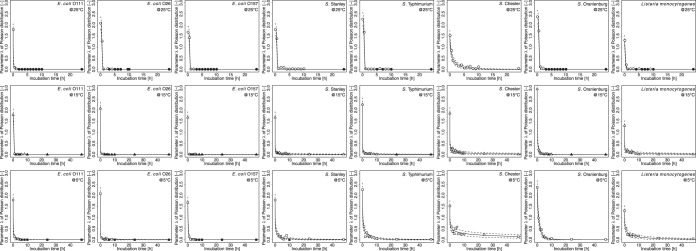
Changes in parameter λ (a unitless measurement [−]) of the Poisson distributions of the numbers of surviving bacteria at 5°C (□), 15°C (△), and 25°C (○) in a desiccated environment. Solid lines, fitted exponential function; dotted lines, uncertainty in the observed outcomes described as the 95% confidence interval of the mean value generated from the Poisson distribution; filled symbols, all the bacteria were dead.

**FIG 2:**
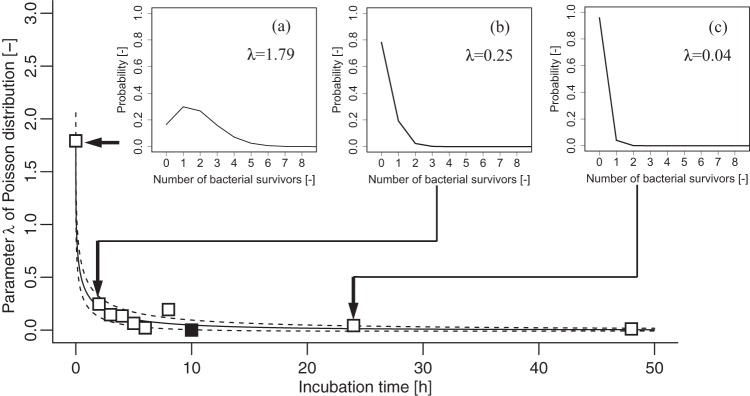
Representative example of the changes in parameter λ of the Poisson distribution of the numbers of surviving bacteria. The data represent changes in the variability in the number of surviving Salmonella enterica serotype Stanley cells at 5°C in a desiccated environment. Solid line, fitted exponential function; dotted lines, variability in the observed outcomes described as the 95% confidence interval of the mean value generated from the Poisson distribution; filled symbols, all the bacteria were dead. The inset graphs (a to c) indicate the probability distributions of the parameter λ of the Poisson distribution, which represent the variability in the number of surviving bacteria.

To describe the variability in the number of surviving bacteria with a Poisson distribution, we fitted a Poisson distribution to the bacterial number data during the bacterial death process using equation 2 (see Materials and Methods) and estimated the value of parameter λ of the Poisson distribution. Changes in the parameter λ of the Poisson distribution are described as an exponential function of the incubation time (Fig. 1). The root mean square error was below 0.095 under all conditions. There was no apparent dependency in the parameters, regardless of the bacterial strains examined (Table 1). Figure 2 shows a representative detailed result of the changes in the survival rate of S. enterica serotype Stanley. The Poisson distributions (λ values) in Fig. 2 illustrate the variability in the number of surviving bacteria at a specific time. The changes in the probability distribution of the numbers of surviving bacterial cells at the single-cell level during the bacterial death process were estimated as Poisson distributions (Fig. 1 and 2). The model developed in the present study enabled us to estimate the changes in the distribution of bacterial numbers.

**TABLE 1:** Exponential fitted survival parameters for λ^*a*^

Strain	*b*			*n*		
	5°C	15°C	25°C	5°C	15°C	25°C
Escherichia coli O111	1.14	1.62	1.97	0.41	2.58	0.52
Escherichia coli O26	1.44	1.96	2.28	0.21	0.24	3.41
Escherichia coli O157:H7	1.10	2.31	2.20	0.79	0.11	5.00
Salmonella Stanley	0.72	1.24	1.63	0.34	0.18	3.87
Salmonella Typhimurium	0.64	1.41	2.02	0.39	0.18	3.95
Salmonella Chester	0.45	0.90	0.48	0.21	0.19	0.59
Salmonella Oranienburg	0.39	1.10	1.33	0.72	0.46	3.29
Listeria monocytogenes	0.48	0.86	2.09	0.33	0.19	1.34

a The parameter λ is calculated as follows: 10^−*bt*^*n*^ + *C*^, where *b* is the survival rate, *t* is the incubation time, *n* is the curvature of the kinetics of the Weibull distribution, and *C* is the initial bacterial distribution.

### Computer simulation using random number generation for bacterial inactivation.

**FIG 3:**
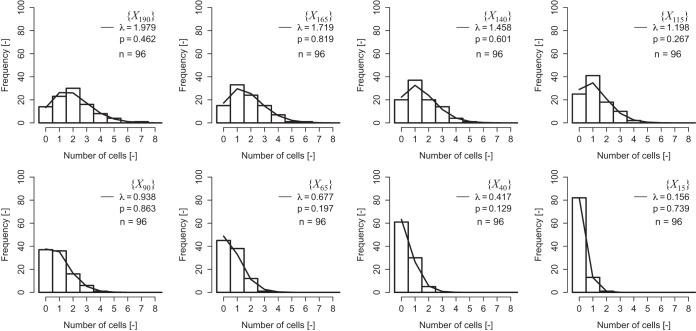
Representative examples of computer simulations of bacterial inactivation using random number generation by use of the Poisson process. Ninety-six random numbers generated from a Poisson distribution (λ = 2) were examined as the initial distribution {*X_N_*}. The distributions of survivors in 96 wells obtained from the simulation are illustrated as histograms. The mean value of the number of survivors in a 96-well microplate is described as parameter λ of the Poisson distribution (solid lines). The *P* values were estimated using a likelihood ratio test. If the *P* value was less than 0.05, the hypothesis that the bacterial cell counts followed a Poisson distribution was rejected.

We conducted a computer simulation to determine whether the frequency distribution of the 96 sets of bacterial numbers followed a Poisson distribution during the bacterial death process in a Poisson process (Fig. 3). Data for the 96 sets of bacterial cells following a Poisson distribution (λ = 2, 10, and 100) were examined as initial state {*X_N_*}, where *N* is the computer-generated total number of bacterial cells. *X_i_* represents the state space consisting of *i* surviving bacteria in a 96-well microplate (*i* = *N*, *N* − 1, ..., 1). As shown in Fig. 3, a *P* value of <0.05 from the likelihood ratio test indicated that the hypothesis that the bacterial cell counts followed a Poisson distribution was rejected. We confirmed that among 1,000 simulations more than 82% of the simulated bacterial distributions (λ = 2) of each state {*X_N_*, *X_N_*_− 1_, … , *X*_1_} followed a Poisson distribution during bacterial death. This result indicates that the distribution of surviving bacterial cells in a small population or at the single-cell level during the bacterial death process follows a Poisson distribution in the Poisson process. The simulated bacterial distribution (λ = 2) also followed a binomial or negative binomial distribution of more than 81%. These two distributions also describe the simulated bacterial distribution well. For the slightly high number of 10 cells, the simulation indicated that more than 80% of the cells followed a Poisson distribution, a binomial distribution, or a negative binomial distribution during the inactivation process. In contrast, for an initial number of 100 cells, less than 55% followed a Poisson distribution, a binomial distribution, or a negative binomial distribution. The mathematical description developed in this study will enable estimation of the probability of bacterial survival with small numbers of cells (e.g., 10 cells) along with its variability.

## DISCUSSION

Previous studies have reported that in small populations, such as those comprising <100 cells, the behavior of individual cells can be very different from the overall behavior of the population (5, 9). These previous studies have described variability from a biological perspective to be mainly the heterogeneity of individual cells, such as differences in tolerance to environmental stresses, such as heat, acidity, or desiccation. Here, we described variability to be the number of survivors based on mathematical descriptions. The focus of the present study was to describe the naturally occurring randomness of small numbers of bacteria on the basis of the assumption that the variability in the numbers of surviving bacteria followed a Poisson distribution. To assume a Poisson process, a Poisson distribution was used to describe variability in the number of surviving bacteria. As a result, the inevitable randomness of small numbers of surviving bacteria in the determination of the numbers was described successfully as the variability in the number of surviving bacteria.

We confirmed whether the distribution of survivors followed a Poisson, binomial, or negative binomial distribution. All three distributions fit well more than 80% of the simulated distributions with an initial state of 2 or 10 cells. Because the mean values for the targeted bacterial cell numbers were very small (2 or 10), a binomial distribution would be very similar to a Poisson distribution (21). In addition, when the sampling numbers for the negative binomial distribution were sufficiently increased (i.e., *n* = 96), the relationship between the mean and variance of a negative binomial distribution is similar to that of a Poisson distribution (21). Thus, it can be concluded that the distribution of survivors is reasonably described as a Poisson distribution with small numbers of cells. The Poisson distribution is more useful than the other two distributions because the Poisson distribution is described by one parameter, λ.

The distinction in the behavior of a small number of bacterial cells compared with that of a larger bacterial population can be attributed to the law of large numbers; that is, as the sample size increases, the mean is more likely to approach the population average. However, to the best of our knowledge, the naturally occurring randomness of bacterial behavior that would represent variability in bacterial cell numbers has not been investigated in detail in a bacterial inactivation process to date. In this study, the variability was assumed to be the naturally occurring randomness in bacterial numbers. Given that the occurrence of such randomness cannot be determined as a fixed value, it was described as a frequency distribution. Here, the variability in the number of surviving bacteria, which represents a naturally occurring random phenomenon, was described as a Poisson distribution. The model developed here enabled estimates of the randomness of bacterial numbers to reflect the realistic nature of random behavior.

The growth behavior of bacterial cells at the single-cell level can be monitored via optical density measurements and/or under a microscope. To estimate the variability in cell growth, single-cell growth models have been developed (9, 22–26). However, there have been few studies in which these dynamics were monitored during the bacterial death process, owing to technical difficulties. Other than viable count methods, it is difficult to distinguish live cells from dead cells. One of the promising techniques for evaluating bacterial cell death processes is a time-lapse microscopic observation method (27). However, a special apparatus is needed for observation, and the procedure limits the applicable objective because the observation must take place while the cells are in a liquid environment. The sampling experiment examined in this study is technically easy to perform and more convenient to handle than monitoring of cell death. Therefore, to effectively and easily estimate changes in the probability of bacterial cell death during the bacterial death process, a sampling evaluation approach would be an effective alternative.

However, it is a challenge to obtain single cells, given the statistical variability and uncertainty associated with the experimental techniques (28, 29). To overcome these technical difficulties with the collection of single cells, we modeled the bacterial numbers as a Poisson distribution, which is a natural distribution that can be easily reproduced (13). Thus, we described the number of surviving bacterial cells as a Poisson distribution rather than as a point estimate because the behavior of these survivors cannot be determined deterministically. Description of the numbers of surviving bacteria as a distribution rather than as the point estimates used in a deterministic approach can provide a more realistic estimate of risk.

Furthermore, we confirmed via computer simulation the variability in the number of surviving bacteria during the bacterial death process at the single-cell level (Fig. 3), which can be associated with the naturally random processes observed in many situations. The probability model developed here can be used to estimate the randomness of the bacterial numbers observed from the perspective of deterministically unexpected bacterial behavior. The variability in bacterial numbers, as defined in this study in the context of desiccation, and the concept of the random occurrence of surviving bacteria could be applied to other processes used to inactivate or kill bacteria, such as those involving heat, pressure, acidity, and irradiation. Probability models that describe the variability in the number of surviving bacteria as a probability distribution can be expected to play an important role in estimating the quantitative risk of bacterial survival.

## MATERIALS AND METHODS

### Bacterial strains.

The bacterial strains were provided by the Research Institute for Microbial Diseases (RIMD) of Osaka University in Japan, the Hokkaido Institute of Public Health (HIPH) in Japan, and the Aomori Prefectural Research Laboratory of Public Health in Japan. One strain each of E. coli O111 (RIMD 05092013), E. coli O26:H11 (RIMD 05091997), E. coli O157:H7 (RIMD 05091897), *S*. Stanley (RIMD 1981001), *S*. Typhimurium (RIMD 1985007), *S*. Chester (from Aomori), *S*. Oranienburg (from Aomori Prefecture), and Listeria monocytogenes (ATCC 13932) was used. All the strains were maintained at −80°C in brain heart infusion broth (Merck, Darmstadt, Germany) containing 10% glycerol. A sterile metal loop was used to transfer the frozen bacterial cultures by scratching the surface of a frozen culture and placing it into 5 ml of tryptic soy broth (TSB; Merck, Darmstadt, Germany) in a sterile plastic tube. The cultures were incubated without agitation at 35°C for 24 h and were transferred, using a loop inoculum, at two successive 24-h intervals to obtain a more homogeneous and stable cell population. Grown cells were collected by centrifugation (1,000 × *g*, 10 min at 25°C), and the resulting pellet was washed twice with peptone water and subsequently resuspended in 5 ml of peptone water.

### Preparation of single cells.

The procedure used for the preparation single bacterial cells was that of Koyama et al. (13). Briefly, single cells of each pathogenic bacterium were prepared by the use of 10-fold dilutions of bacterial cultures. The initial cell count was assumed to be approximately 10^9^ CFU/ml. The inoculum was further diluted (10-fold dilution series) in peptone water to obtain a solution with 10^3^ CFU/ml, and the bacterial cell number was determined via direct plating of 100 μl of the inoculum of a sample with 10^3^ CFU/ml onto five tryptic soy agar plates, which were then incubated at 35°C for 24 h. The data are expressed as the mean value (*x̄*) for five replicates of plate counts. An aliquot of the solution with 10^4^ CFU/ml stored from the previous day of the experiment was diluted *x̄*/10-fold in pure water to obtain a solution with 1,000 CFU/ml. Pure water was used to prevent the bacteria from being stressed by the high osmotic pressure used during desiccation. This procedure resulted in a final solution of 2 CFU/2 μl.

### Assumption of a stochastic bacterial death process.

In the present study, we assumed that the bacterial death process followed a Poisson process. An occurrence or event was independent of itself, and occurrences or events appeared one by one over an infinite time period. The variability in the total number of occurrences or events until a specific time period follows a Poisson distribution in a Poisson process (21, 30). According to the nature of a Poisson process, we experimentally investigated and computationally simulated bacterial death.

### Bacterial inactivation in a desiccated environment.

Single cells of each bacterial serotype in 2 μl of solution were dispensed into each well of 96-well microplates. The prepared microplates were dried in a drying chamber using silica gel (10% to 20% relative humidity) at 5, 15, and 25°C. We confirmed the survival or death of the cells in each well by adding 100 μl of TSB about every 1 or 2 h until 10 h and every 24 h until 48 h. One microplate was used to determine the survival or death of cells at each time and under each condition. The microplates to which TSB was added were stored at 25°C for 1 week to allow the recovery of injured bacterial cells (31, 32). The turbidity of the wells was observed to determine the survival or death of cells.

### Analysis of data on the change in the survival rate during the drying process.

We assumed that the number of survivors followed a Poisson distribution during bacterial death. We determined the proportion of wells that contained surviving cells by counting the number of wells in the 96-well plates with surviving bacteria to estimate the survival probability parameter λ of the Poisson distribution. The Poisson distribution is described by equation 1:

(1) $$P(X=k)=\frac{{\text{λ}}^{k}e^{-\text{λ}}}{k!}$$

where *P*(*X = k*) is the probability of finding *k* cells in a randomly chosen well and λ is the parameter of the Poisson distribution.

From the rate of survival determined from wells with surviving bacteria, we estimated the parameter λ of the Poisson distribution. From equation 1, the parameter λ of the Poisson distribution was calculated as follows:

(2) λ =−log[P(X=0)]

Given that the parameter λ of the Poisson distribution was continuous and nonnegative, we used a nonnegative decreasing mathematical function to analyze the data. We described a change in the value of parameter λ of the Poisson distribution over time as an exponential function on the basis of the Weibull distribution by nonlinear regression. The Weibull distribution is commonly used to describe the log reduction (33) and has been used to describe bacterial inactivation kinetics (34, 35). The mean value of the response variable λ, which is the number of survivors, follows a Poisson distribution of mean λ. Equation 3 was used as the basic fitting model:

(3) $$\log_{10}\text{λ}\;={-bt}^{n}+C\;$$

where *b* and *n* represent the survival rate and the curvature of kinetics of the Weibull distribution, respectively, and *C* and *t* are the initial bacterial distribution and the incubation time, respectively, and were estimated by use of the λ parameter. We used equation 3 to estimate the parameter λ of the Poisson distribution as follows:

(4) $$\text{λ}\;=\;{\mathrm{10}}^{{-bt}^{n}\;+\;C}$$

The variability in the observed outcomes was estimated as the variability in the mean value for the 96 sets of bacterial numbers generated from the Poisson distribution. We estimated the variability in the observed outcomes as the 95% confidence interval of the mean value generated from the Poisson distribution.

### Bacterial inactivation simulation using random number generation.

**FIG 4:**
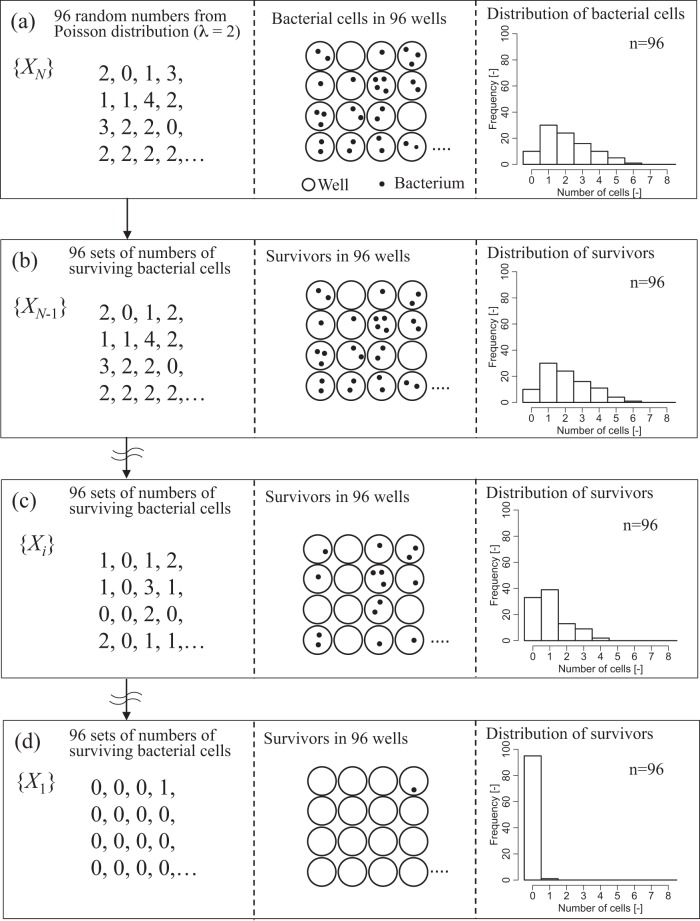
Schematic diagram summarizing the computer simulation for the changes in the frequency distribution of the numbers of surviving bacteria under a Poisson process. {*X_i_*} is a set of the numbers of bacteria in the wells (*i* = *N*, *N* − 1, …, 1). *N* is the total number of bacteria initially in state {*X_N_*}. We confirmed whether the bacterial number in each state ({*X_i_*}) followed a Poisson distribution using a likelihood ratio test. (a) Initial state in which *N* cells survive ({*X_N_*}); (b) state in which *N* − 1 cells survive ({*X_N_*_− 1_}); (c) state in which *i* cells survive ({*X_i_*}); (d) state in which 1 cell survives ({*X*_1_}).

We simulated the stochastic death process for 96 sets of bacterial cells by the generation of random numbers by computer simulation in a Poisson process (Fig. 4). We determined whether the frequency distribution of 96 sets of bacterial cell numbers followed a Poisson distribution during the bacterial death process. The simulation of the bacterial death process was based on the following presuppositions: the time to inactivation of an individual cell is independent of any surrounding cells, and the time to inactivation of individual cells occurs one by one over an infinite time period.

**FIG 5:**
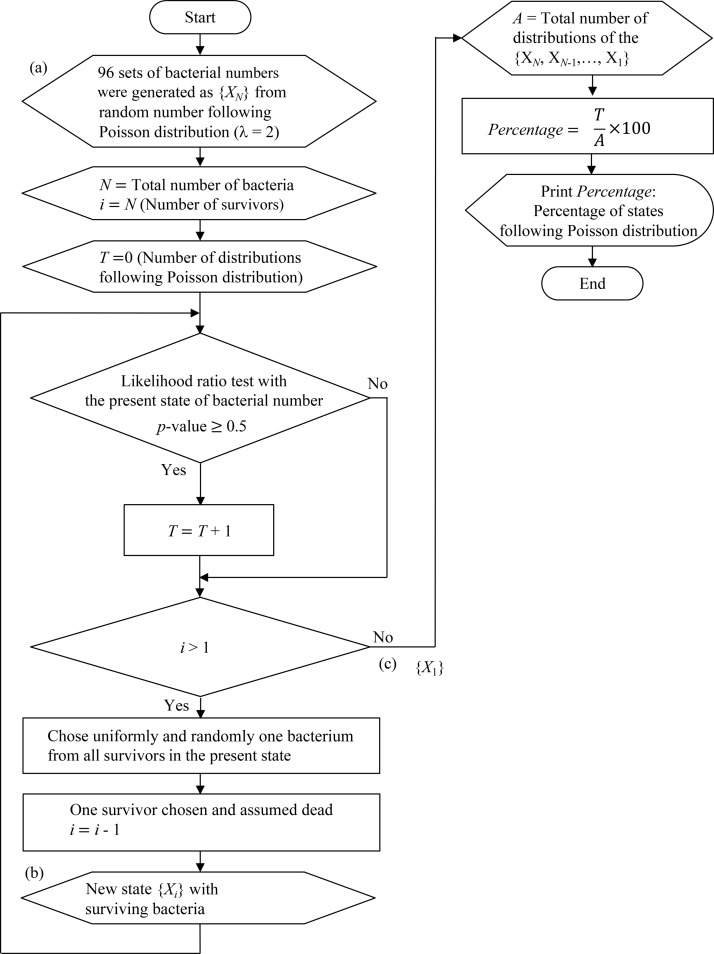
Flowchart of the computer simulation used to change the frequency distribution of the number of surviving bacteria under a Poisson process. *T* is the number of distributions following a Poisson distribution. (a) Initial state in which *N* cells survive; (b) state in which *i* cells survive; (c) state in which 1 cell survives.

The computer simulation examined a bacterial death process from 96 sets of virtual bacterial cells following a Poisson distribution. Ninety-six random numbers following a Poisson distribution (λ = 2) were generated, as shown in Fig. 4a and 5a. {*X_N_*} represents the initial state space consisting of the number of bacteria in a 96-well microplate. *N* is the computer-generated total number of bacterial cells. We uniformly and randomly chose a surviving bacterial cell from {*X_N_*} and assumed it to be a dead cell. The number of survivors then became *N* − 1, and the initial state {*X_N_*} transitioned to state {*X_N_*_− 1_} (Fig. 4b). As the number of survivors decreased one by one, transition of the state occurred until state {*X_i_*} (where *i* = *N*, *N* − 1, …, 1) (Fig. 4c and 5b) became state {*X*_1_}, which contained one surviving cell (Fig. 4d and 5c). The computer simulation was repeated 1,000 times. The algorithm is summarized as a flowchart in Fig. 5. We repeated the computer simulation and examined the bacterial death process from 96 sets of virtual bacterial cells following two different Poisson distributions (λ = 10 or λ = 100).

We determined whether the probability distribution of surviving bacterial cell numbers in each state {*X_N_*, *X*_*N* − 1_, … , *X*_1_} followed a Poisson distribution during the death process using a likelihood ratio test (36). If the *P* value was less than 0.05, the hypothesis that the bacterial cell counts followed a Poisson distribution was rejected. We also determined whether a binomial distribution and a negative binomial distribution fit the probability distribution of surviving bacterial cell numbers in each state.

All statistical analyses were conducted with R statistical software (version 3.1.2 for Mac OS X; http://www.r-project.org).
